# Robotic versus laparoscopic versus open major hepatectomy – an analysis of costs and postoperative outcomes in a single-center setting

**DOI:** 10.1007/s00423-023-02953-x

**Published:** 2023-05-29

**Authors:** Sebastian Knitter, Linda Feldbrügge, Nora Nevermann, Brigitta Globke, Santiago Andres Ortiz Galindo, Thomas Winklmann, Felix Krenzien, Philipp K. Haber, Thomas Malinka, Georg Lurje, Wenzel Schöning, Johann Pratschke, Moritz Schmelzle

**Affiliations:** Department of Surgery, Campus Charité Mitte and Campus Virchow-Klinikum, Charité – Universitätsmedizin Berlin, corporate member of Freie Universität Berlin and Humboldt-Universität zu Berlin, and Berlin Institute of Health, Augustenburger Platz 1, 13353 Berlin, Germany

**Keywords:** Liver surgery, Liver resection, Laparoscopic liver resection, Robotic liver surgery, Robotic-assisted liver surgery, Cost analysis

## Abstract

**Purpose:**

In the era of minimal-invasive surgery, the introduction of robotic liver surgery (RS) was accompanied by concerns about the increased financial expenses of the robotic technique in comparison to the established laparoscopic (LS) and conventional open surgery (OS). Therefore, we aimed to evaluate the cost-effectiveness of RS, LS and OS for major hepatectomies in this study.

**Methods:**

We analyzed financial and clinical data on patients who underwent major liver resection for benign and malign lesions from 2017 to 2019 at our department. Patients were grouped according to the technical approach in RS, LS, and OS. For better comparability, only cases stratified to the Diagnosis Related Groups (DRG) H01A and H01B were included in this study. Financial expenses were compared between RS, LS, and OS. A binary logistic regression model was used to identify parameters associated with increased costs.

**Results:**

RS, LS and OS accounted for median daily costs of 1,725 €, 1,633 € and 1,205 €, respectively (*p* < 0.0001). Median daily (*p* = 0.420) and total costs (16,648 € vs. 14,578 €, *p* = 0.076) were comparable between RS and LS. Increased financial expenses for RS were mainly caused by intraoperative costs (7,592 €, *p* < 0.0001). Length of procedure (hazard ratio [HR] = 5.4, 95% confidence interval [CI] = 1.7–16.9, *p* = 0.004), length of stay (HR [95% CI] = 8.8 [1.9–41.6], *p* = 0.006) and development of major complications (HR [95% CI] = 2.9 [1.7–5.1], *p* < 0.0001) were independently associated with higher costs.

**Conclusions:**

From an economic perspective, RS may be considered a valid alternative to LS for major liver resections.

**Supplementary information:**

The online version contains supplementary material available at 10.1007/s00423-023-02953-x.

## Introduction

Laparoscopic liver surgery (LS) has undergone a rapid development over the last decades and is now considered an established alternative to open liver surgery (OS) [1]. Starting with the first laparoscopic cholecystectomy in 1987 [2], the indication for LS was incrementally extended, eventually including technically demanding posterolateral and major resections. The short-term advantages of LS, including postoperative improvements of morbidity, mortality, and length of hospital stay [3–5], are well known, while oncologic outcomes were reported to be at least equivalent to OS [6–10].

Over the last years, robotic surgery has gained interest in the field of liver surgery, after the robotic platform has been introduced to a variety of surgical domains including colorectal, thoracic and urologic procedures [11–13]. Similar to LS, the first described robotic liver surgery (RS) was a cholecystectomy in 1994 [14]. Since then, the use of RS was described for wedge resections [15], resections of posterosuperior segments [16], hemihepatectomies [17], extended resections of perihilar cholangiocarcinoma [18], and living donor hepatectomies [19]. Apart from frequently quoted surgeon-oriented advantages of RS such as a more extensive range of movement, hand tremor filtration or the three-dimensional view [20–22], known improvements in short-term outcomes of LS over OS have also been reported for RS in comparison to OS including reduction of postoperative morbidity and length of hospital stay [23, 24]. However, RS may be associated with increased costs deriving from high acquisition and maintenance costs, and longer procedure times when compared to OS or LS [25, 26]. By reducing morbidity and length of stay, increased costs for RS are expected to be amortized, though only few recent studies analyzed the cost-effectiveness between OS, LS and RS. These studies found conflicting results: While most authors reported higher costs [27, 28], some quoted comparable [29, 30] or even lower costs for RS [31].

The aim of this study was to compare RS, LS, and OS for major liver resections by evaluating costs in a single-center experience. In addition, we aimed to identify patient-based and perioperative factors that were associated with increased costs.

## Methods

### Patient inclusion criteria and study design

In this single-center retrospective study, data on all consecutive patients who underwent liver surgery from 2017 to 2019 at the Department of Surgery, Campus Charité Mitte and Campus Virchow-Klinikum, Charité – Universitätsmedizin Berlin, were collected. Patients were included regardless of the indication for surgery and, if one of the following procedures were performed: left or right hemihepatectomy and extended left or right hemihepatectomy without biliary or vascular reconstruction. In addition, only cases in the German Diagnosis Related Groups (DRG) H01A (complex operations of the liver and pancreas with complex intensive care treatment) and H01B (operations of the liver and pancreas without complex intensive care treatment) were considered for inclusion aiming to provide a cost evaluation of complex liver surgery. For better comparability of costs, patients who were assigned other DRGs, e. g. due to longer stays on the intensive care unit (ICU) because of organ failure, were excluded. Further exclusion criteria were concomitantly performed procedures (e. g. colostomy reversals), and if patients were aged < 18 years at time of resection. Patients were then stratified according to the form of surgery in RS, LS, and OS. Approval for this study was obtained from the ethics commission of our institution (EA2/006/16 and EA4/084/17).

### Perioperative management and surgical procedures

A standardized preoperative evaluation protocol including medical history with physical examination, laboratory tests, and an anesthesia evaluation was conducted for each patient. Cross-sectional imaging (triphasic contrast-enhanced computed tomography and/or magnetic resonance imaging, if needed with liver-specific contrast agents) was performed depending on the indication of surgery. For malign diagnoses, each case was presented at the multidisciplinary tumor board of our institution providing a recommended individual treatment strategy. The decision between RS, LS and OS was made on an individual basis, and was dependent on patient-related factors, such as previous abdominal surgery, comorbidities and individual preference, and on the surgeon’s discretion.

Surgical procedures were performed as previously described [32]. A modified Makuuchi incision initiated OR [33]. For RS and LS, patients were kept in the French position [34]. In case of LS, the following techniques were performed: multiport laparoscopic surgery with a transumbilical 12 mm optical trocar and further 5 mm and 12 mm trocars or hand-assisted laparoscopic surgery using a hand port via a 5 cm supraumbilical incision and 2–3 additional 12 mm ports. For RS, the DaVinci Xi® Surgical System (Intuitive Surgical Inc., Sunnyvale, CA, USA) was used as previously described by our group [35, 36]. Four 8 mm DaVinci® trocars were placed on an imaginary line 2 cm below the inferior border of the liver at intervals of 7 cm. Up to three additional assist trocars (2 × 12 mm and 1 × 5 mm) were used for laparoscopic assistance by the table-site surgeon.

Regardless of RS, LS or OS, each procedure was started with an examination of the peritoneal cavity to rule out any undiagnosed tumor spread. The exact location of benign or malign lesions of the liver were validated using intraoperative ultrasound. In LS, the following devices were used for parenchymal dissection: energy devices (Thunderbeat®, Olympus K.K., Tokyo, Japan, or Harmonic Ace®, Ethicon Inc., Somerville, NJ, USA), laparoscopic cavitron ultrasonic surgical aspirator (CUSA®, Valleylab Boulder, CO, USA), Waterjet (ERBEJET®, ERBE Tübingen, Germany), and vascular stapler (Echelon™, Ethicon, Somerville, NJ, USA) or Endo GIA™ (Medtronic, Dublin, Ireland). In RS, *da Vinci Xi HARMONIC ACE* Curved Shears and in OS CUSA® (Valleylab Boulder, CO, USA) were used. Intermittent hilar occlusion was used for hepatic vascular exclusion, as needed. For RS and LS, resected specimens were retrieved via a Pfannenstiel incision or an extended median umbilical incision.

Postoperatively, patients were admitted to our specialized surgical intensive care unit (ICU), if needed. Nasogastric tubes were removed on the day of surgery and early oral intake was planned for the same postoperative day. Intraoperatively placed abdominal drains were removed early on, as soon as the discharge was quantitively und qualitatively unremarkable. Postoperative morbidity and mortality were defined as any complication or death within 90 days after surgery. The classification of Clavien and Dindo was used to grade postoperative complications, and major morbidity were defined as grade ≥ 3a [37]. Patients were closely monitored for postoperative complications such as intra-abdominal bleeding, infection, biliary fistula, pulmonary complications, or liver failure by regular clinical visitations, blood tests, and imaging methods, if needed.

Regardless of benign or malign lesions, all specimens were evaluated by the Department of Pathology. In case of malign lesions, all cases were re-assessed in the interdisciplinary tumor board, and a further treatment was recommended.

### Cost analysis

The controlling department of our center routinely collects financial data of all cases and provided these data for this study. Apart from the total expenses per case, costs were generally categorized into the following groups: (1) surgery, (2) anesthesia, (3) ICU, (4) normal ward, (5) laboratory tests, (6) radiology including interventions such as computer tomography-guided drainages, (7) endoscopy for therapeutic interventions, (8) other diagnostics, (9) other therapeutics (e. g. physiotherapy), and (10) patient admission. Intraoperative costs included expenses for medical staff, consumables such as surgical devices (staplers, clips, etc.) and operating room time per minute. Daily costs were calculated by dividing total costs and length of stay. All numbers were presented in Euro (€).

### Statistical analysis

Continuous variables were expressed as medians (range), and categorical variables were presented as frequencies. All data was compared between the three groups (RS, LS, OS) using the Chi square test for categorical variables and the Kruskal–Wallis *H* test for continuous variables. When significant, individual group comparisons were performed using the Chi square, Fisher’s exact or Mann Whitney *U* test as appropriate. A binary logistic regression model was used to identify factors associated with increased costs, which was defined for all cases with higher costs than the 75^th^ percentile. Parameters with *p* < 0.1 in univariate analysis (Chi square or Fisher’s exact test) were entered in the multivariate analysis, and results were expressed as hazard ratio (HR) and 95% confidence interval (CI).

*P* values < 0.05 were considered statistically significant. SPSS software package, version 25 (IBM, Armonk, NY, USA) was used to perform statistical analyses.

## Results

### Patient characteristics

During the study period, 146 patients who underwent major liver resection at the Department of Surgery, Campus Charité Mitte and Campus Virchow-Klinikum, Charité – Universitätsmedizin Berlin could be identified according to the inclusion and exclusion criteria. Among the excluded cases were 61 patients, who were assigned other DRG groups than H01. RS, LS, and OS were performed in 25 (17%), 59 (40%), and 62 (43%) patients.

Patient characteristics are presented in Table 1. Gender distribution was significantly different with predominantly men in the OS group (*p* = 0.002). However, comparing RS and LS, gender distribution was comparable (*p* = 0.165). No statistical differences could be found for other baseline characteristics such as median age at resection (*p* = 0.136), median BMI (*p* = 0.631), American Society of Anesthesiologists (ASA) score ≥ 3 (*p* = 0.850), or indication for surgery (*p* = 0.702 for benign, and *p* = 0.132 for malign diagnoses).

**Table 1:** Patient characteristics of 146 patients who underwent major liver resection

Characteristics	RS (*n* = 25)	LS (*n* = 59)	OS (*n* = 62)	*P*^*1*^	*P*^*2*^	*P*^*3*^	*P*^*4*^
Sex, *n* (%)				0.002	0.165	0.001	0.013
Female	16 (64)	28 (48)	16 (26)				
Male	9 (36)	31 (52)	46 (74)				
Median age at resection, years (range)	61 (27–79)	57 (26–83)	65 (29–81)	0.136	-	-	-
Age ≥ 65, years, *n* (%)	12 (48)	23 (39)	32 (52)	0.369	-	-	-
Median BMI, in kg/m^2^ (range)	24 (18–38)	25 (18–41)	25 (17–45)	0.631	-	-	-
BMI ≥ 30, kg/m^2^, *n* (%)	4 (16)	9 (15)	16 (26)	0.302	-	-	-
ASA score ≥ 3, *n* (%)	9 (36)	21 (36)	25 (40)	0.850	-	-	-
Cases per year, *n* (%)				< 0.0001	< 0.0001	< 0.0001	0.475
2017	0 (0)	17 (29)	12 (19)				
2018	0 (0)	23 (39)	27 (44)				
2019	25 (100)	19 (32)	23 (37)				
Dignity, *n* (%)				0.072	-	-	-
Benign	8 (32)	11 (19)	7 (11)				
Malign	17 (68)	48 (81)	55 (89)				
Benign diagnoses, *n* (%)				0.702	-	-	-
Adenoma	2 (25)	4 (36)	0 (0)				
Hemangioma	1 (13)	1 (9)	1 (14)				
Cystic disease	0 (0)	0 (0)	0 (0)				
Caroli syndrome	3 (38)	1 (9)	2 (29)				
Chronic cholestasis or cholangitis^1^	1 (13)	2 (18)	1 (14)				
Traumatic lesions	0 (0)	1 (9)	1 (14)				
Abscess	0 (0)	0 (0)	0 (0)				
Echinococcosis	0 (0)	2 (29)	2 (29)				
Other benign diagnoses^2^	1 (13)	1 (9)	0 (0)				
Malign diagnoses, *n* (%)				0.132	-	-	-
Metastatic disease^3^	1 (6)	6 (13)	2 (4)				
Colorectal liver metastases	4 (24)	21 (44)	18 (33)				
HCC	3 (18)	13 (27)	14 (26)				
Cholangiocarcinoma	9 (53)	8 (17)	20 (36)				
Other malignant diagnoses^4^	0 (0)	0 (0)	1 (2)				
Extent of hepatic resection, *n* (%)				< 0.0001	< 0.0001	0.047	< 0.0001
Right hemihepatectomy, *n* (%)	5 (20)	32 (54)	18 (29)				
Extended right hemihepatectomy, *n* (%)	4 (16)	8 (14)	21 (34)				
Left hemihepatectomy, *n* (%)	7 (28)	18 (31)	5 (8)				
Extended left hemihepatectomy, *n* (%)	9 (36)	1 (2)	18 (29)				

*P*^1^, all; *P*^2^, RS vs. LS; *P*^3^, RS vs. OS; *P*^4^, LS vs. OS; ICU, intensive care unit; BMI, body mass index; ASA, American Society of Anesthesiologists; ^1^ including primary sclerosing cholangitis; ^2^ including focal nodular hyperplasia; ^3^ excluding colorectal liver metastases; ^4^ including sarcoma and lymphoma

### Perioperative data

Clinical outcome variables are presented in Table 2. Median lengths of procedure were comparable between all groups (*p* = 0.867). Postoperative morbidity was 40%, 29%, and 48% (*p* = 0.087), and postoperative major morbidity was observed in 16%, 14%, and 29% (*p* = 0.090), for RS, LS, and OS, respectively. Postoperative bile leakage was evident in 16%, 9%, and 15% of cases after RS, LS, and OS (*p* = 0.497). Patients were admitted to the ICU for a median length of one day in all groups (*p* = 0.090), with a trend towards shorter stays after RS (maximum length of 2 days in comparison to 8 and 6 days after LS and OS, respectively). Median length of hospital stay was longest after OS with 14 days vs. 9 days for RS and LS, respectively (*p* < 0.0001). Between RS and LS, the lengths of stay were equivalent (*p* = 0.947). There was no postoperative mortality in all groups.

**Table 2:** Perioperative clinical data of 146 patients who underwent major liver resection

Parameters	RS (*n* = 25)	LS (*n* = 59)	OS (*n* = 62)	*P*^*1*^	*P*^*2*^	*P*^*3*^	*P*^*4*^
Median length of procedure, minutes (range)	316 (180–457)	313 (98–605)	298 (99–621)	0.867	-	-	-
Median length of stay on ICU, days (range)	1 (0–2)	1 (0–8)	1 (0–6)	0.071	-	-	-
Median length of hospital stay, days (range)	9 (7–31)	9 (6–44)	14 (6–71)	< 0.0001	0.947	0.016	< 0.0001
Postoperative overall morbidity, *n* (%)	10 (40)	17 (29)	30 (48)	0.087	-	-	-
Postoperative major morbidity, *n* (%)	4 (16)	8 (14)	18 (29)	0.090	-	-	-
Postoperative bile leakage, *n* (%)	4 (16)	5 (9)	9 (15)	0.497	-	-	-
Postoperative mortality, *n* (%)	0 (0)	0 (0)	0 (0)	-	-	-	-

*P*^1^, all; *P*^2^, RS vs. LS; *P*^3^, RS vs. OS; *P*^4^, LS vs. OS; ICU, intensive care unit

### Analysis of costs after RS, LS and OS

**Fig. 1:**
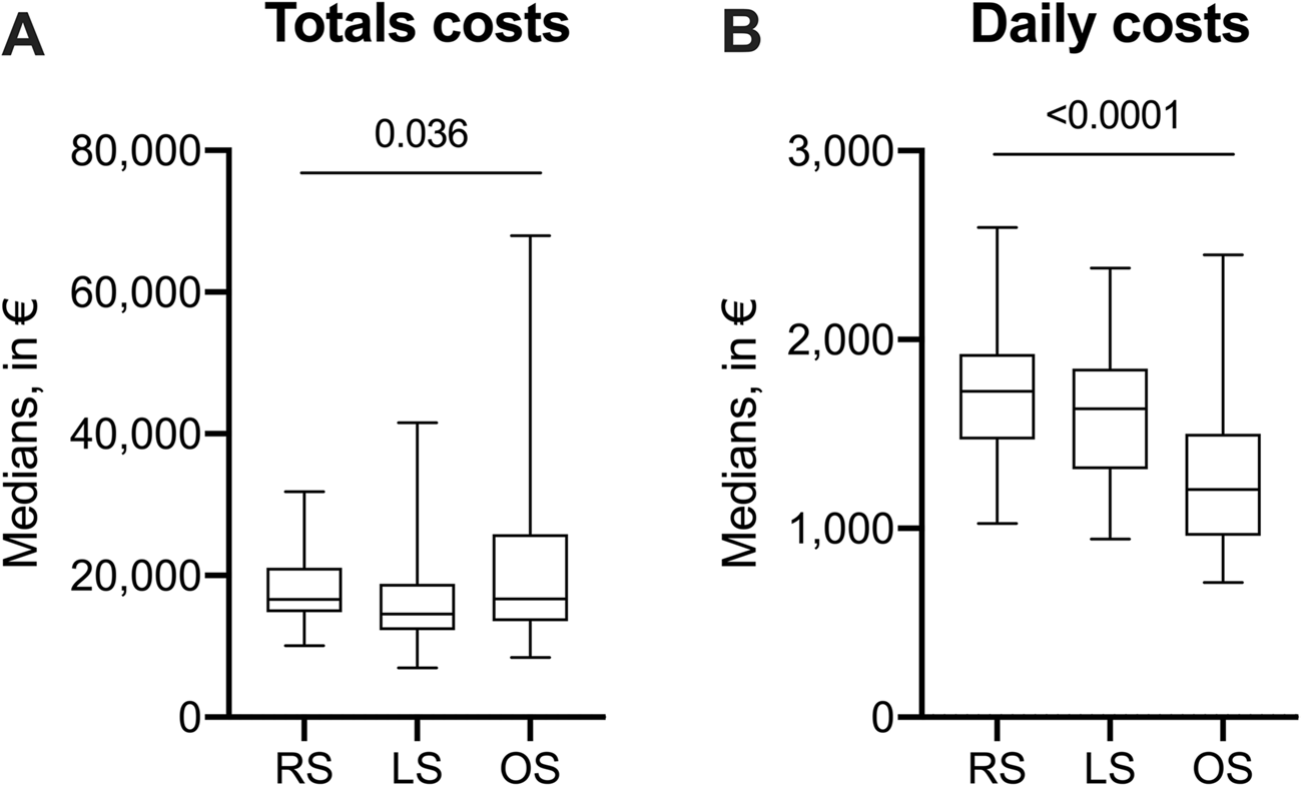
Median (**A**) total and (**B**) daily costs for RS, LS and OS of 146 patients who underwent major liver resection

Financial expenses differed significantly for several categories (see Table 3). Median total costs were highest after OS in comparison to RS and LS (16,701 € vs. 16,648 € vs. 14,578 €, *p* = 0.036; see Fig. 1). Post-hoc analysis revealed no differences between RS and LS (*p* = 0.076), and RS and OS (*p* = 0.815). Median total costs after LS were significantly lower than after OS (*p* = 0.016). Median daily costs were highest after RS with 1,725 €/day (*p* < 0.0001), however, the difference between RS and LS was not significant in post-hoc analysis (*p* = 0.420; see Fig. 1). Several differences could be found between the individual items: Most notably, expenses for surgery were highest for RS (7,592 € per case) and lowest for OS (4,500 € per case; *p* < 0.0001). Comparing RS and LS (6,387 €/case), the significant difference persisted (*p* = 0.002). Other differences include costs for normal ward care, which was lowest for LS (3,614 €, *p* < 0.0001), and for laboratory tests, which was highest for OS (1,541 €, *p* < 0.0001).

**Table 3:** Cost analysis of 146 patients who underwent major liver resection

Parameters	RS (*n* = 25)	LS (*n* = 59)	OS (*n* = 62)	*P*^*1*^	*P*^*2*^	*P*^*3*^	*P*^*4*^
Surgery, median, € (range)	7,592 (3,745–13,962)	6,387 (2,466–11,616)	4,500 (1,493–16,276)	< 0.0001	0.002	< 0.0001	0.001
Anesthesia, median, € (range)	2,393 (1,578–3,138)	2,360 (732–3,457)	2,618 (1,285–9,768)	0.128	-	-	-
ICU, median, € (range)	708 (0–2,516)	707 (0–6,771)	793 (0–5,731)	0.085	-	-	-
Normal ward, median, € (range)	3,802 (2,174–11,123)	3,614 (1,910–18,807)	5,666 (1,800–33,155)	< 0.0001	0.973	0.001	< 0.0001
Laboratory tests, median, € (range)	1,122 (478–2,163)	1,058 (417–4,443)	1,541 (485–3,979)	< 0.0001	0.996	0.001	< 0.0001
Radiology, median, € (range)	156 (0–1,904)	242 (0–1,609)	401 (0–4,266)	0.061	-	-	-
Endoscopy for therapeutic interventions, median, € (range)	1,788 (74–2,991)	1,211 (1,020–2,138)	2,016 (592–8,859)	0.399	-	-	-
Other diagnostics, median, € (range)	43 (0–230)	39 (0–316)	69 (0–574)	0.019	0.568	0.010	0.081
Other therapeutics, median, € (range)	113 (0–261)	89 (0–446)	142 (0–1,397)	0.009	0.503	0.068	0.008
Patient admission, median, € (range)	0 (0–278)	0 (0–193)	0 (0–549)	0.985	-	-	-
Median daily costs, € (range)	1,725 (1,026–2,593)	1,633 (944–2,380)	1,205 (714–2,450)	< 0.0001	0.420	< 0.0001	< 0.0001
Median total costs, € (range)	16,648 (10,092–31,815)	14,578 (6,982–41,548)	16,701 (8,421–67,964)	0.036	0.076	0.815	0.016

*P*^1^, all; *P*^2^, RS vs. LS; *P*^3^, RS vs. OS; *P*^4^, LS vs. OS; ICU, intensive care unit

Cost analysis of excluded cases that were assigned other DRG groups can be found in the Supplementary Data (Supplementary Table 1).

### Factors associated with increased total costs

Aiming to identify factors that are associated with increased total costs for all patients, a multivariate analysis of baseline and perioperative parameters was conducted (see Table 4). Increased total costs were defined as higher total expenses than the 75^th^ percentile per group accounting for 21,144 €. The following parameters raised overall costs as indicated by univariate analysis: malignant diagnosis (79% vs. 92%, *p* = 0.087), length of procedure (*p* < 0.0001), length of hospital stay (*p* < 0.0001), postoperative overall (27% vs. 75%, *p* < 0.0001) and major morbidity (8% vs. 58%, *p* < 0.0001), and postoperative bile leakage (6% vs. 33%, *p* < 0.0001). Multivariate analysis revealed the following factors to be independently predictive of higher costs: longer duration of surgery (hazard ratio [HR] = 5.4; confidence interval [CI] = 1.7–16.9; *p* = 0.004), longer length of hospital stay (HR = 8.8; CI = 1.9–41.6; *p* = 0.006), and postoperative major morbidity (HR = 2.9; CI = 1.7–5.1; *p* < 0.0001).

**Table 4:** Multivariate analysis of factors associated with increased total costs in 146 patients who underwent major liver resection

Parameters	UV			MV	
	< 21,144 € per case (*n* = 110)	≥ 21,144 € per case (*n* = 36)	*P*	HR (95% CI)	*P*
Male sex, *n* (%)	63 (57)	23 (64)	0.484		
Age ≥ 65 years, *n* (%)	50 (46)	17 (47)	0.853		
BMI ≥ 30 kg/m^2^, *n* (%)	19 (17)	10 (28)	0.170		
ASA score ≥ 3, *n* (%)	39 (36)	16 (44)	0.334		
Malignant diagnosis, *n* (%)	87 (79)	33 (92)	0.087		NS
Length of procedure, *n* (%)					
≥ 308 min	43 (39)	30 (83)	< 0.0001	5.4 (1.7–16.9)	0.004
Postoperative ICU stay, *n* (%)	98 (89)	35 (97)	0.187		
Length of hospital stay, *n* (%)					
≥ 10 days	45 (41)	34 (94)	< 0.0001	8.8 (1.9–41.6)	0.006
Postoperative morbidity, *n* (%)	30 (27)	27 (75)	<0.0001		NS
Postoperative major morbidity, *n* (%)	9 (8)	21 (58)	< 0.0001	2.9 (1.7–5.1)	< 0.0001
Postoperative bile leakage, *n* (%)	6 (6)	12 (33)	<0.0001		NS

UV, univariate analysis; MV, multivariate analysis; BMI, body mass index; ASA, American Society of Anesthesiologists; ICU, intensive care unit; NS, not significant

## Discussion

The steady increase in financial healthcare expenses warrants analyses of costs and value, which is particularly relevant for new equipment and technologies. In case of minimally invasive liver surgery, concerns about the financial burden have been raised throughout the history of its introduction and establishment [38, 39]. In this study, we provide an analysis of costs after RS, LS, and OS for major liver resections in a single-center experience. Our data showed that median total costs were comparable between RS (16,648 €) and OS (16,701 €), and lowest after LS (14,578 €), without a statistical difference between RS and LS (*p* = 0.076). Median daily costs were highest after RS (1,725 €/day), however, no statistical difference between RS and LS could be found (*p* = 0.420). As main cost driver, highest intraoperative costs were observed for RS (7,592 € per case, *p* < 0.0001).

Using multivariate analysis, we identified longer procedures, longer hospital stays, and the development of postoperative major complications to be independently associated with increased costs. Importantly, short-term postoperative outcomes including length of procedures, length of ICU stay, postoperative overall and major morbidity, and postoperative bile leakage were comparable for RS, LS and OS. Only the length of hospital stay was significantly different between the groups and longest after OS (14 days, *p* < 0.0001), but equivalent after RS and LS (9 vs. 9 days, *p* = 0.947). These results confirm that RS is a safe procedure and may be considered as an alternative for LS.

Several recent meta-analyses compared the postoperative course of RS with LS and OS and mainly found longer procedure lengths for RS while other postoperative outcome variables such as complication rates were equivalent [25, 40–42]. In our study, we reported comparable rates of postoperative overall and major morbidity, and postoperative mortality, in this highly selected population of patients who underwent major liver surgery and were assigned the DRG H01. Procedure lengths were equivalent between all groups in our study, and length of stay did not differ between RS and LS. Still, a non-significant trend towards better postoperative outcomes could be observed after LS in comparison to RS, which was evident by lowest rates of postoperative morbidity and bile leakage in the LS group. The difference between RS and LS may be attributed to the learning curve of RS, as it was introduced to our clinic during the study period in 2019 [43–45].

High acquisition and maintenance costs may hinder the widespread establishment of RS [26]. Hence, several studies evaluated the associated costs of RS in comparison to LS and OS giving conflicting results. A recent retrospective analysis by Aziz et al. analyzed readmission rates and costs based on the United States National Readmission Database with one of the largest populations so far, and found lower readmission rates and lower costs for RS in comparison to both LS and OS [46]. In contrast, Miller et al. reported higher costs for RS compared to LS based on a database by the American College of Surgeons, which was related to blood transfusions, lengths of procedure and hospital stay [47]. Similarly, most studies quoted higher costs for RS [27, 28, 48], while others reported comparable [29, 30] or even lower costs indicating better cost-effectiveness for RS [31]. Recent meta-analyses summarized available studies and concluded higher expenses for RS in comparison to LS [25, 49, 50]. Our data confirm these results as median total costs were higher after RS than after LS (16,648 € vs. 14,578 €, *p* = 0.076). However, the difference was non-significant. Furthermore, we tried to provide further insight in the composition of costs by compiling a point-by-point analysis. As expected, the primary driver of increased costs for RS were expenses related to the surgery itself (7,592 €, *p* = 0.002). In contrast to OS, intraoperative costs for minimal-invasive procedures are mainly raised by special laparoscopic devices [51] and longer operation times.

Next, we aimed to identify drivers of higher costs in our population by using a multivariate analysis. Length of procedure, length of stay, and postoperative major morbidity were independently associated with higher costs. All these parameters are known to be cost-extensive for hospitals, and especially major complications add a significant financial burden [52]. However, major morbidity was not significantly different in our groups, and therefore did not influence financial results. In addition, these findings may explain the cost differences for individual items as seen in our point-by-point analysis, which identified highest costs for normal ward care and laboratory tests for OS. Both items highly depend on the length of the hospital stay, and the development of postoperative complications.

Our study also has several limitations. First, evidence was acquired in a retrospective manner and was derived from only one center, therefore results may be prone to selection bias, which may have been further aggravated by our focus on the DRG group H01. By excluding other DRG groups, for example those for patients with longer ICU stays due to severe complications, more cost-extensive cases were left out. Since postoperative major morbidity and length of stay were identified as independently associated with increased costs, cases from other DRGs may strongly influence these results. In addition, some baseline characteristics were significantly different between our groups, which may have impacted the analyses. Namely, gender distribution varied between the groups, and more patients with benign lesions underwent RS. In addition, one must assume a learning curve effect for robotic, but also for laparoscopic surgery in this period, which will be reflected in shorter operating times and a lower complication rate over the following years. After completion of the learning curve, a more balanced distribution of costs may be possible. Still, we believe that our data contributes to the ongoing discussion concerning the cost-effectiveness of RS. Importantly, we present the first cost evaluation of robotic liver surgery in a German high-volume center.

## Conclusions

For major liver resection, RS is associated with a financial burden that is comparable to LS. Patient selection remains crucial, and further studies of high-volume centers of liver surgery in DRG-based countries are needed to determine the cost-effectiveness of the robotic approach.

### Supplementary information

Below is the link to the electronic supplementary material.

Supplementary file1 (DOCX 16.4 KB) 
