# Supplementary material for: Robotic versus laparoscopic versus open major hepatectomy – an analysis of costs and postoperative outcomes in a single-center setting
Source: Langenbecks Arch Surg. 2023 May 29;408(1):214. doi: 10.1007/s00423-023-02953-x (PMC10226911; doi:10.1007/s00423-023-02953-x)
Supplement: Supplementary file 1 — Supplementary file1 (DOCX 16.4 KB) [file 423_2023_2953_MOESM1_ESM.docx]

**Supplementary Data**

**Supplementary Table 1.** Cost analysis of 61 patients of other DRG groups who underwent major liver resection

| **Parameters** | **LS**  **(n = 14)** | **OS**  **(n = 47)** | ***P*** |
| --- | --- | --- | --- |
| Surgery, median, € (range) | 9,134  (4,835-20,475) | 8,066  (2,556-35,359) | 0.824 |
| Anesthesia, median, € (range) | 4,556  (1,658-7,095) | 3,789  (1,438-17,435) | 0.932 |
| ICU, median, € (range) | 10,854  (661-39,159) | 23,554  (0-177,638) | **0.045** |
| Normal ward, median, € (range) | 8,214  (1,272-22,648) | 9,612  (534-39,525) | 0.797 |
| Laboratory tests, median, € (range) | 2,989  (1,266-8,688) | 5,735  (1,159-55,230) | **0.040** |
| Radiology, median, € (range) | 1,774  (50-8,469) | 2,296  (49-14,068) | 0.904 |
| Endoscopy for therapeutic interventions, median, € (range) | 1,137  (0-4,473) | 411  (0-7,366) | 1 |
| Other diagnostics, median, € (range) | 103  (11-944) | 122  (0-453) | 0.719 |
| Other therapeutics, median, € (range) | 484  (37-1,016) | 991  (54-3,756) | **0.006** |
| Patient admission, median, € (range) | 0  (0-193) | 0  (0-322) | 0.690 |
| Median daily costs, € (range) | 1,532  (686-2,656) | 1,565  (769-3,607) | 0.391 |
| Median total costs, € (range) | 41,946  (17,905-89,062) | 59,499  (18,536-284,671) | 0.053 |

ICU, intensive care unit
